# Pulmonary Cement Embolism following Percutaneous Vertebroplasty

**DOI:** 10.1155/2014/851573

**Published:** 2014-12-14

**Authors:** Ümran Toru, Tuba Coşkun, Murat Acat, Hilal Onaran, Şule Gül, Erdoğan Çetinkaya

**Affiliations:** ^1^Department of Chest Diseases, Faculty of Medicine, Dumlupınar University, 43100 Kütahya, Turkey; ^2^Department of Chest Diseases, İstanbul Florence Nightingale Hospital, 34381 Istanbul, Turkey; ^3^Department of Chest Diseases, Faculty of Medicine, Karabük University, 78100 Karabük, Turkey; ^4^Department of Chest Diseases, Merzifon Karamustafa Paşa State Hospital, 05300 Amasya, Turkey; ^5^Department of Chest Diseases, Yedikule Chest Diseases and Thoracic Surgery Training and Research Hospital, 34020 Istanbul, Turkey

## Abstract

Percutaneous vertebroplasty is a minimal invasive procedure that is applied for the treatment of osteoporotic vertebral fractures. During vertebroplasty, the leakage of bone cement outside the vertebral body leads to pulmonary cement embolism, which is a serious complication of this procedure. Here we report a 48-year-old man who was admitted to our hospital with dyspnea after percutaneous vertebroplasty and diagnosed as pulmonary cement embolism.

## 1. Introduction

Percutaneous vertebroplasty (PV) is an interventional radiologic procedure that involves injection of polymethylmethacrylate (PMMA) bone cement into a cervical, thoracic, or lumbar vertebral body lesion for the relief of pain and the strengthening of bone in symptomatic vertebral compression fractures [1].

Passage of bone cement into the venous system and then inferior vena cava, subsequently, into the lungs is one of the rare and serious complications of PV [1, 2].

We present a case of a 48-year-old man who was admitted to our hospital with dyspnea on postoperative first day of PV and diagnosed as pulmonary cement embolism (PCE).

## 2. Case Report

**Figure 1:**
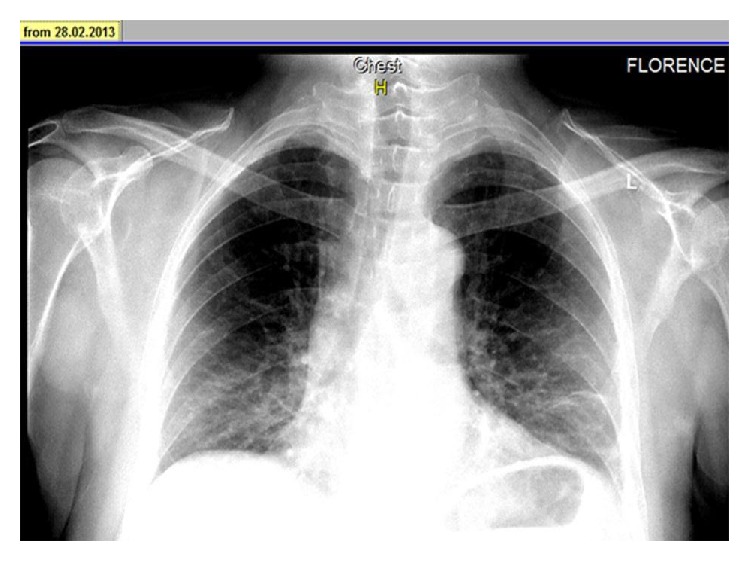
PA radiography showed linear densities in the left lower zone and elevation of the left diaphragm.

**Figure 2:**
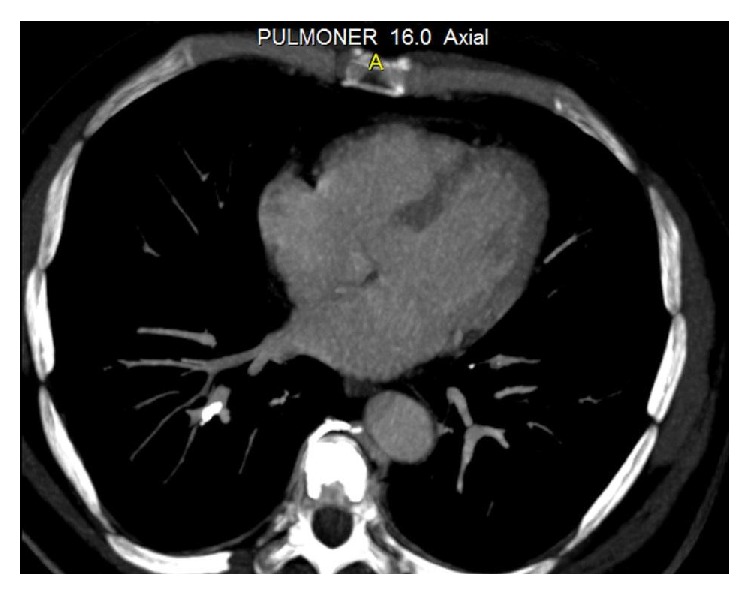
Cement leakage to the azygos vein was detected in thorax CT.

**Figure 3:**
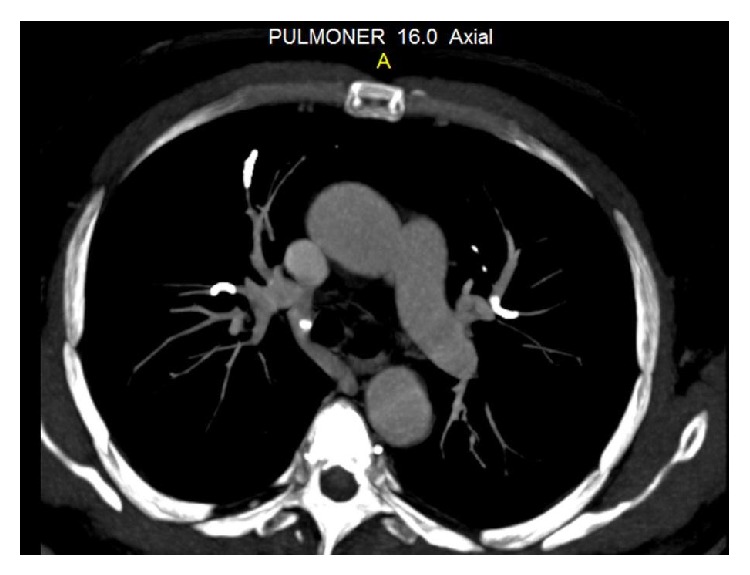
Axial view of thorax CT showing multiple, linear hyperdensities in bilateral lobar pulmonary artery branches.

**Figure 4:**
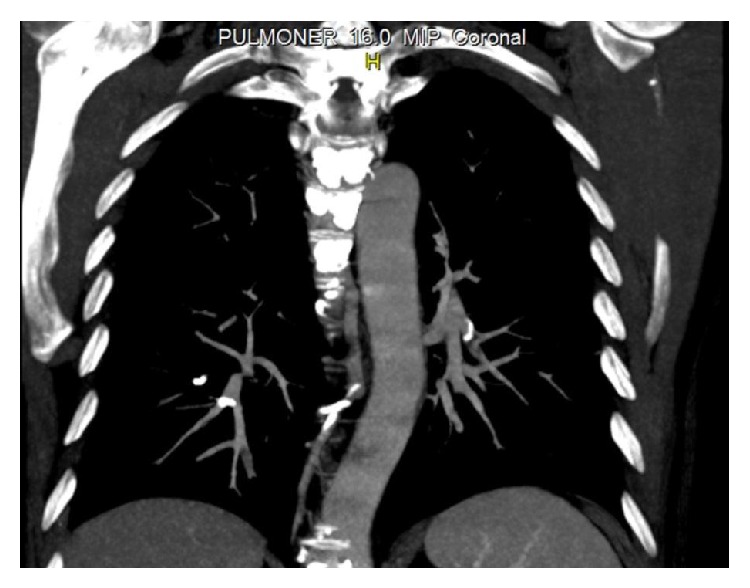
Coronal view of thorax CT.

A 48-year-old man was admitted to our hospital with a complaint of dyspnea, which occurred within the first 24 hours after vertebroplasty. His anamnesis revealed the use of corticosteroids for six months with the diagnosis of chorioretinitis. Because of iatrogenic osteoporosis, he underwent vertebroplasty with the diagnosis of an osteoporotic vertebral fracture in another hospital. Posteroanterior (PA) chest radiography showed linear densities that are consistent with linear atelectasis in the left lower zone and elevation of the left diaphragm (Figure 1). Consecutively, computed tomography (CT) of thorax was performed with a prediagnosis of pulmonary embolism. Thorax CT revealed the cement leakage to the azygos vein (Figure 2) and also multiple bilateral, linear hyperdensities within the lobar pulmonary artery branches are detected in axial and coronal views of CT (Figures 3 and 4). Size and function of the right ventricle were within normal limits in echocardiography (ECO). Considering the patient's recent vertebroplasty, we attributed these clinical and radiological findings to pulmonary embolization of the bone cement, which was used during the procedure. The patient was treated with low molecular weight heparin (LMWH) and antibiotherapy. We did not observe any problems in clinical follow-up of the patient who continued LMWH treatment for 3 months.

## 3. Discussion

PV is a widely applied therapeutic approach for symptomatic vertebral compression fractures, especially those of osteoporotic origin, and for osteolytic vertebral tumors [3, 4]. During this procedure, PMMA bone cement is injected into the vertebral body transcutaneously under CT or image guidance [4]. Potential complications of PV include bleeding at the puncture site, pain, fever following injection, bone infection or fracture, nerve or spinal cord damage, radiculopathy or paralysis, leakage of bone cement into the epidural or paravertebral spaces, and passage of cement into the venous system with embolization to the pulmonary vasculature [1].

The only risk factor identified for the development of PCE is fluoroscopic evidence of cement leakage to the azygos vein or vena cava during vertebroplasty [5, 6]. Bone cement extravasates toward the vertebral venous plexus, which is connected to the azygos system. By this way, cement reaches the inferior vena cava and then the pulmonary arterial system, which results in PCE [2, 7].

In 38 to 73% of cases, cement leaks into the perivertebral tissue but remains silent in most cases [2, 8, 9]. Only in 1% of osteoporotic fractures, this leakage causes clinical symptoms [8]. Clinical indicators of PCE are sudden occurrence of symptoms such as dyspnea, tachypnea, tachycardia, cyanosis, chest pain, cough, hemoptysis, and sweating after vertebroplasty [10]. The only symptom of our patient was dyspnea, which occurred within the first 24 hours of PV.

Since most cases are initially asymptomatic, many cement emboli are found incidentally on subsequent imaging. That is why routine chest radiography is recommended after vertebroplasty [3, 11, 12]. In X-rays, cement emboli may be seen as multiple tubular or branched radiopacities [13]. Thorax CT confirms the presence of bone cement in the branches of pulmonary arteries. Thus, performance of CT scans for early detection of cement emboli in the pulmonary circulation is recommended too [13]. ECO is a useful method for assessing the development of pulmonary hypertension in patients with symptomatic or multiple emboli [13]. In our case, we primarily performed PA radiography and then CT to reveal the etiology of dyspnea. PA chest radiography showed linear densities in the left lower zone and thorax CT confirmed the presence of multiple linear hyperdensities in bilateral lobar pulmonary artery branches. Also, we performed ECO to observe the right ventricular function and pulmonary arterial pressure. According to these clinical and radiological findings, our diagnosis was PCE following PV.

Treatment of PCE reduces the risk of thrombus formation, pulmonary embolism, pulmonary infarction, and respiratory failure. In the literature, treatment is recommended based on the severity of symptoms, location, and size of the pulmonary embolism [13, 14]. From this point of view, the patients with PCE can be divided into 4 groups: (1) asymptomatic peripheral embolism, (2) symptomatic peripheral embolism, (3) asymptomatic central embolism, and (4) symptomatic central embolism [15]. No treatment is recommended for asymptomatic patients with peripheral embolisms [10]. Conservative treatment and regular clinical follow-up are the therapy choices for this group [15]. In cases of symptomatic peripheral or asymptomatic central embolisms, it is recommended to follow standard treatment guidelines for pulmonary thromboembolism, which include initial heparinization (i.v. or s.c.) followed by 3 to 6 months of warfarin therapy [10, 15]. In those patients with symptomatic central embolisms, surgical treatment with embolectomy is suggested [11, 16]. Our case was consistent with the symptomatic peripheral embolism group. We started treatment with LMWH and continued for 3 months. Although we did not use warfarin, there were not any problems in our clinical follow-up.

## 4. Conclusion

Patients with respiratory symptoms after vertebroplasty should be evaluated carefully in terms of pulmonary cement embolism.
